# Treatment of Diseases of the Larynx

**Published:** 1880-10

**Authors:** E. Fletcher Ingals

**Affiliations:** Lecturer on Diseases of the Chest and Physical Diagnosis, and on Laryngology in the Post Graduate Course, Rush Medical College


### ARTICLE II.

**TREATMENT OF DISEASES OF THE LARNYX.** By E. FLETCHER INGALS, M.D., Lecturer on Diseases of the Chest and Physical Diagnosis, and on Laryngology in the Post Graduate Course. Rush Medical College.

GENTLEMEN:—Having pointed out in my recent lectures the symptoms and signs which will enable you to diagnosticate diseases in the larnyx, I wish this morning to call your attention to the methods of treatment for the most common diseases, which may be best employed by the general practitioner.

### IN ACUTE LARYNGITIS, if the patient is brought to us sufficiently early, our first effort should be to abort the attack; for this purpose it is customary to give at bedtime ten grains of Dover's powder, or from six to ten grains of quinine, or a hot "sling," either one of which will frequently enable the patient to rise in the morning comparatively well; or, instead of these, aconite may be given, or the fluid extract of jaborandi; either will answer much the same purpose as the Dover's powder. Theoretically, jaborandi should be a very valuable remedy in such cases, but generally I have failed to get a good article when I have prescribed it. In a few cases it has acted very satisfactorily, but in others the discomfort which attends its physiological action has been very great, on account of the salivation, and often nausea and vomiting.

A gentleman of my acquaintance once took half a drachm of the fluid extract, and went immediately to bed, hoping to fall asleep before the diaphoresis began. He describes the effects as follows: "In about twenty minutes I felt my mouth fill with saliva, and on rising to expectorate it perspiration started from every pore; my mouth was scarcely emptied before expectoration was again necessary, and this continued until the act became so tiresome that I was obliged to hang my head over the edge of the bed and allow the saliva to run in a steady stream; the sweat poured from every part of my body almost in streams, and saturated the bed clothing; this continued for about three hours, the monotony varied only by occasional vomiting, and at about the end of two hours a cessation in the flow of saliva, which, however, promptly returned when I moved to arrange myself for sleep."

This remedy might doubtless be taken in the daytime without proving so uncomfortable as at night, when the patient wants to sleep.

Failing to abort the attack, you will next resort to remedies which diminish the fever and lessen the inflammation or favor its speedy resolution. With this in view the bowels should be gently acted on by saline cathartics, and you may administer opiates internally in small and repeated doses, giving about one thirtieth of a grain of morphia or its equivalent every half hour, or less frequently, or you may give grain doses of compound ipecac powder, or small doses of other narcotics. Aconite may be given with good results in doses of half a drop every fifteen minutes for a couple of hours, subsequently diminishing the frequency, and increasing the size of the dose to one or two drops every two hours. In view of the danger from œdema, some are in favor of early administering a free purge of calomel, and subsequently using the mercurial in small and frequent doses. The inhalation of steam, or steam impregnated with volatile anodynes, is usually beneficial. The atmosphere of the room should be kept moist, and at a temperature of about 70° F. or 75° F.

With children, the steam from a kettle may be kept playing near the mouth, and older persons may inhale from the spout of a teapot filled with warm water, or from an ordinary steam atomizer. When the patient inhales the vapor from warm water, the addition of some anodyne is desirable, such as one drachm of compound tincture of benzoin, or the same with two to five drops of chloroform, or eight grains of the extract of conium, or a drachm of extract of lupulin rubbed up with half a drachm of carbonate of sodium and a little water, and added to the pint of water at 150° F., or remedies may be administered by the steam atomizer advantageously, as, for instance, two or three grains of carbolic acid to half an ounce each of glycerine and water, or mild astringent solutions with or without small quantities of opium or belladonna. For these you may employ a couple of grains of sulphate of zinc to an ounce of water, or tannin in about the same proportions, or acetate of lead.

If there is much pain in the throat, or if the person is restless, the amount of opium given internally may be increased, or you may add some of the watery extract of opium 'or belladonna to the solution which is used for atomization; in many cases the camphorated tincture of opium, or the compound tincture of benzoin, acts very pleasantly, whether used as a vapor in the warm water or as a spray by the steam atomizer.

Strong topical applications cannot be made by the general practitioner, and are of doubtful efficacy, even when properly applied. Strong solutions of the mineral astringents, especially from sixty to one hundred and twenty grains of nitrate of silver, are highly extolled by some physicians, but in the majority of instances patients seem to get along better without them.

If œdema of the larnyx supervenes, the case tends to a fatal issue unless promptly checked or relieved. The best method of treatment is scarification of the mucous membrane so as to allow escape of the serum which has collected in the submucous tissues. Scarification of the epiglottis and occasionally the superior portions of the larnyx can usually be performed by the general practitioner by the aid of a long bistoury, around which has been wrapped, to within a quarter of an inch of its point, a piece of adhesive plaster for the purpose of avoiding injury to the tongue.

By the aid of the laryngoscope we are enabled to scarify the deeper portions of the larynx more carefully and effectually. This is the best treatment, but where it cannot be practiced the administration of emetics is sometimes followed 'by good results, due to the act of vomiting which occasionally causes rupture of the mucous membrane.

The fluid extract of jaborandi might be given in some of these cases with benefit. In one instance in which I employed it the œdema was greatly relieved. If efforts to relieve the œdema fail, and the obstruction to respiration increases, tracheotomy will become necessary.

Frequently a subacute form of inflammation follows acute laryngitis, and may continue for a long time. This will require the application of stronger remedies. The general physician may apply them by means of an atomizer with a long tube properly bent to throw a spray into the throat, the patient being directed to respire deeply, or to sound a prolonged, high-pitched note during the application. For this purpose almost any of the mineral astringents may be used in moderately strong solutions, or various astringent powders may be thrown into the larynx, but these latter are not so desirable as topical applications of strong astringents by means of a brush. For application with a brush or sponge we may use sulphate or chloride of zinc, twenty or thirty grains to the ounce of water, or water and glycerine, or twenty grains of the perchloride or persulphate of iron. One hundred and twenty grains of sulphate of iron, ten grains of sulphate of copper, or twenty to thirty grains of alum to the ounce of fluid, may be used in a similar manner. Some physicians prefer nitrate of silver in solutions varying in strength from twenty to even one hundred and twenty grains, but the other astringents seem to act equally as well, and are less likely to be painful. Counter irritation at the upper part of the sternum or upon the back of the neck will sometimes be found useful.

ACUTE LARYNGITIS IN YOUNG CHILDREN

requires more vigorous treatment than in adults, because of the small size of the larynx, and the greater liability to spasm of the glottis. In treating these cases the warm bath should be used at first to relieve the engorgement of the mucous membrane and tendency to spasm. The atmosphere of the room should be kept moist by steam, and the temperature kept up to 80° F. or 85° F., and when possible the little patient should be induced to inhale steam from the atomizer. Frequently young children become very much alarmed by the atomizer, when brought close to their faces, but they will get some benefit from it though it is placed three or four feet away. A great deal of benefit will frequently be derived from warm applications, care being taken to keep the parts constantly warm and moist. For this purpose poultices of flaxseed are as good as anything, or you may use cloths wrung out of hot water, or spongiopilin with warm water which latter is an elegant application; whichever of these is employed it must be kept constantly hot, for if allowed to cool it will do more harm than good. If these cannot be kept warm it is much better to apply dry cloths. Turpentine stupes to the neck have also been found beneficial. If there is much tendency to spasm, the compound syrup of squills may be given, or small doses of belladonna, which not only relieve the spasmodic tendency, but possibly have some specific curative effect on the mucous membrane of the throat.

If œdema comes on, you should make an effort to scarify the part, but generally this cannot be effected in young children; failing in this, by passing the finger over the base of the tongue, you will sometimes be able to tear the mucous membrane with the nail, and thus allow the serum to escape. If you cannot relieve the œdema, and the dyspnœa continues to increase, do not hesitate to resort to tracheotomy, which holds out very good chances for recovery.

In a few rare instances of acute laryngitis in young children, the dyspnœa seems to be due to inflammation of the posterior crico-arytenoid muscles, which are the abductors of the vocal cords. The glottis during respiration in health is a triangular chink, but with paralysis of these muscles the cords are drawn together during inspiration, so as to greatly interfere with the ingress of air. In one case of this sort, reported by Dr. J. Solis Cohen, it was found that the application of ice bags to the neck every minute for about eight hours succeeded in inducing reflex respiratory movements, which carried the child over the critical period.

IN SUB-ACUTE LARYNGITIS, which is the form of affection found in ordinary colds, the treatment is generally attended to by the patient himself; if consulted, you should manage the case as one of mild laryngitis, taking care to prevent exposure to cold, keeping the patient in his room, and applying the milder remedies recommended for the acute affection.

PHLEGMONOUS LARYNGITIS

is a very grave affection, and therefore calls for more vigorous treatment. Early in the disease, the application of leeches along the edge of the sternum, or in the inter-clavicular notch, has been recommended, and you should at once have recourse to fomentations and the inhalation of steam, more or less impregnated with opium or belladonna according to the amount of distress. Subsequently, the case should be treated upon the supporting and stimulating basis, consisting of nourishing diet, alcoholics, and quinine and iron. The diet should be fluid, and, as the patient will ordinarily be unable to swallow, it must generally be given by enema. Quinine may be given hypodermically, or by enema, if the patient cannot swallow. In this disease, scarification is of little benefit. Tracheotomy is indicated when the patient suffers much dyspnœa, but it is usually fruitless.

IN ERYSIPELATOUS LARYNGITIS

the inhalation of steam is beneficial, and when impregnated with anodynes it will add much to the patient's comfort. Frequently emetics are useful in young children, for the sake of clearing the throat. The most desirable emetic in such cases is alum or the sulphate of copper; alum is usually the best, but neither of them causes subsequent prostration. Benefit has also been obtained in these instances, by causing the patient to hold small bits of ice in his mouth.

Some specialists have found benefit from the topical use of very strong solutions of nitrate of silver.

Tracheotomy is indicated if dyspnœa becomes urgent, but usually it will not save the patient.

TRAUMATIC LARYNGITIS

is an affection which any of you are likely to be called upon to treat, especially that form resulting from the inhalation of steam by young children. The most satisfactory treatment consists of the inhalation of steam more or less impregnated with substances which relieve the smarting, and anodynes used internally or hypodermically, to relieve the pain and restlessness. At the same time the patient should be well nourished, and stimulation may very soon be found necessary. The constant application of bags of ice to the neck, and sucking bits of ice, are also beneficial. Considerable relief from the smarting in the mouth and throat is obtained by the inhalation of atomized solutions of the acetate of lead, or carbonate of soda. Mucilaginous drinks, as, for instance, flaxseed tea, or barley water, will also be found beneficial. These drinks are frequently very distasteful to the patient, but if acidulated with a little lemon juice, they become quite palatable.

Calomel is recommended in doses of two or three grains once an hour, until relief is obtained. I dislike to give it, but it has the sanction of high authority.

Œdema comes on very soon, and if it should cause much obstruction to respiration, tracheotomy must not be delayed, for if it is, the chance of success will be much less, or the patient may suddenly die from suffocation. The œdema seldom extends below the glottis, therefore the operation is generally successful if performed sufficiently early; but if the blood is allowed to become surcharged with carbonic acid, the relief afforded does not come soon enough to prevent the cerebral or pulmonary complications which are likely to result from the congestion.

ABSCESSES OF THE LARNYX

are likely to cause death by asthenia or apnœa. The treatment should be supporting from the first, and if possible to reach the abscess, it should be opened; but if this cannot be done tracheotomy should be performed as soon as the dyspnœa becomes urgent.

ŒDEMA OF THE LARYNX.

In this affection ice bags applied to the neck or ice held in the mouth will give considerable relief, and tannin applied in spray, powder, or by the brush, will be found beneficial, but scarification is the most rational treatment, and it has the advantage of giving immediate and usually permanent relief. Chronic œdema is not much improved by scarification, because it usually depends upon a fibrinous instead of a serous exudation. The application of strong mineral astringents is likely to give some relief.

IN SUB-GLOTTIC ŒDEMA, occurring just beneath the vocal cords, the various topical applications are almost useless. Usually the œdema is due to a fibrinous exudation, so that even if scarification could be practiced, it would be without result. In these cases dyspnœa can only be relieved by tracheotomy, and subsequently, in the majority of cases, the tube must be constantly worn.

### CROUP

may be beneficially treated in much the same manner as acute laryngitis. The patient should be kept in a room with moist atmosphere, at a temperature of about 85°. Hot fomentations may be applied to the neck ; emetics are valuable to clear the throat of mucus, and occasionally they aid in detaching false membrane, but only those should be used which have no depressing effects, such as alum, sulphate of copper, or turpeth mineral. Iron and quinine may be needed. Chlorate of potassium seems to have a good effect in some instances, and in others, small doses of the permanganate of potassium have given very satisfactory results. The insufflation or internal use of sulphur has been greatly lauded by the laity recently, and has been tried very generally by the profession, apparently with some success. Inhalations of the vapor from slacking lime, or atomized lime water are often very useful in promoting the disintegration and detachment of the false membrane.

The patient should take these inhalations every twenty minutes or half-hour. Vapor from slacking lime may be obtained by dropping bits of lime into a basin of hot water and holding over it a piece of paper rolled into the form of a funnel, which will collect the vapor and direct it into the patient's mouth, or the basin may be placed near the patient's head, and both be covered with an apron or sheet.

Lime water may be used with the hand atomizer, or better still with the ordinary steam atomizer. If this treatment does not answer the purpose, you should try a solution of one grain of bromine and five to sixty grains of bromide of potassium to the ounce of water, used in the same manner as the lime water; or the two may be used alternately.

The tendency to spasm which nearly always exists in true croup should be met by the administration of opium or belladonna, which will render the disease a little less dangerous. Considerable benefit seems to have been derived in some cases from the application of bags of ice to the neck.

In croup, the results of tracheotomy are not nearly so satisfactory as in acute laryngitis, or œdema following injury to the throat; however, the operation should be resorted to in case dyspnœa becomes urgent. The chances of success will be greatly increased by an early operation; therefore as soon as there is marked lividity of the lips and falling in of the soft parts of the chest during inspiration, tracheotomy should be performed; for unless relief is obtained, death is certain. Be sure of the diagnosis, and then operate early, and you may hope to save from twenty to fifty per cent. of your patients. Late operations are not nearly as successful, and without tracheotomy, about ninety-five per cent. of well-marked cases of croup are fatal.

During convalescence from croup, the patient should be confined to the house for several weeks, and great care be taken that the body is not chilled by changes in the temperature or clothing. If the patient is allowed to go out, a recurrence of the attack is quite probable.

I lately saw a little child, in consultation, who was, at the time, almost comatose from obstruction to the respiration by croupy exudation. The remedies which were being employed, aided by the inhalation of vapors of lime water, had such a beneficial effect that on the following morning the child seemed almost well. The next day the people allowed it to play in the street, and the next day it was buried.

After convalescence from croup, the voice frequently remains hoarse for a number of weeks or months; this is due to subacute inflammation, paresis of the vocal cords, or perhaps to the formation of morbid growths within the larynx. Time usually effects a cure.

### LARYNGISMUS STRIDULUS: SPASMODIC OR CEREBRAL CROUP.

During the paroxysm, an attempt should be made to relax the spasm by slapping the back, buttock, etc., dashing cold water in the face, cold sponging, or by the warm bath. After the paroxysm, you must attempt to remove the cause of the spasm, which may be found in the alimentary canal, prepuce, spinal cord, or brain. If you find swollen gums, lance them; if an overloaded stomach, give emetics; if you find the bowels irritated by undigested food, give a dose of castor oil; if there is phimosis and irritation of the glans, circumcise; or if the cause seems seated in the brain or spinal cord, give bromide of potassium.

The affection in children is usually associated with slight catarrhal laryngitis, and the spasm is perhaps most frequently excited by an overloaded condition of the stomach. In such cases, turpeth mineral in doses of one-fourth to one-half grain, or the compound syrup of squills in doses of fifteen to thirty drops every fifteen minutes until vomiting is produced, and subsequently moderate doses of bromide of potassium or oxide of zinc and extract of hyoscyamus, is a prompt and efficient course of treatment. Following the attack, small doses of belladonna will be useful. Iron, cod liver oil, etc., are indicated to improve the general condition.

This disease, especially in adults, is occasionally due to paralysis of the posterior crico-arytenoid muscles, which allows the vocal cords to fall together during inspiration and thus obstruct the entrance of air. In cases of this sort, if they are at all persistent, tracheotomy should be performed to remove the immediate danger, and allow the patient time to recover from the cause of the paralysis. After tracheotomy, the application of electricity, internally or externally, to the larnyx, would seem to be indicated, but it has not proven very successful. The administration of tonics, including strychnia, is usually followed by good results.

CHRONIC LARYNGITIS

is usually a difficult disease to manage, because the treatment must be prolonged and the patient is very likely to become discouraged. In mild cases, by attending to the general health, placing your patient under as good hygienic conditions as possible, and administering such simple tonics or laxatives as may seem indicated, you will be likely to get a good result without topical applications. A cure may often be hastened by causing the patient to inhale the spray, two or three times a day, of a mild solution of some vegetable or mineral astringent. Tannin is the most common remedy for this purpose, but sulphate of zinc is a little more pleasant. It should be used in the proportion of about two grains to the ounce of water; or we may employ in the same manner sulphate of copper, one or two grains; or acetate of lead, two to five grains to the ounce. These remedies are to be used when the amount of secretion is considerable. When the mucous membrane is dry and the secretion scant, we will find good results from a similar application of iodide of potassium, five grains; chloride of ammonium, five or ten grains; or the tincture of pyrethrum, ten minims to the ounce. Water is generally used as the menstruum, though we may use a mixture of glycerine and water. The nascent chloride of ammonium may be employed by any one of the numerous inhalers devised for the purpose.

When chronic laryngitis is attended by pain, the camphorated tincture of opium, or the compound tincture of benzoin, or the watery extract of opium or belladonna, may be beneficially added to the astringent spray. Patients who are being treated by inhalations of warm vapors or steam sprays must not go out of doors for half or three-quarters of an hour after the inhalation.

In case the disease does not yield to this form of treatment, sulphate of zinc, chloride of zinc, or the sulphate of copper, ten to thirty grains to the ounce; sulphate of iron, from sixty to one hundred and twenty grains; or nitrate of silver, thirty to one hundred and twenty grains to the ounce, or some other astringents or caustics, should be applied directly to the inflamed parts with the aid of the laryngoscope and laryngeal brush or sponge. Ordinarily, these strong applications should be made at first every day, for one or two weeks, then every second day, and afterwards less frequently. In the meantime the patient should use at home some of the milder astringent sprays of which I have just spoken. When the patient is obliged to breathe cold air, or air containing irritating dust, the use of a respirator to modify the temperature or filter the air will be found very beneficial.

In some instances the best treatment which can be applied will be found inefficient; then you will have to be content with palliative measures and maintaining the general health in good condition. All cases must receive proper constitutional treatment besides the local applications. Such patients are frequently benefited by change of climate.

When ulceration occurs in simple chronic laryngitis or in the syphilitic or tuberculous forms, the treatment must be more vigorous. Syphilitic ulcers should be treated by topical applications of the solid nitrate of silver, or very strong solutions; or the acid nitrate of mercury, forty to one hundred and twenty grains to the ounce; or when the ulceration is on the epiglottis, so that it can be reached, the galvano-cautery may prove more efficient. The applications will not need to be repeated oftener than once in two or three days. In the ulcerations due to a simple chronic laryngitis, the same topical applications are indicated as in chronic inflammation without ulceration, but the solution should be stronger and should be applied carefully to the ulcerated surface. In tuberculous laryngitis with ulceration, and dysphagia due to pain, very great benefit may be derived from local applications. For the general practitioner, the simplest local remedies are cod liver oil or sweet oil. When swallowed they bathe the parts more or less completely, and thus aid in preventing the severe pain which would otherwise prevent the ingestion of food. The same remedies may be applied with the brush or probang. The insufflation of certain powders will be found beneficial, and this may usually be easily accomplished by the general practitioner. The insufflator which I have found most satisfactory consists of a rubber bulb attached to a flexible tube about eighteen inches in length, and a glass tube about eight inches in length and three-sixteenths of an inch in diameter, bent within an inch of its extremity nearly to a right angle and made somewhat flaring at the orifice of this bent end. The insufflators found at the instrument stores consist of a metal or gutta-percha tube, bent at one extremity and having a rubber bulb attached to the other. The objection to these is that as the bulb is squeezed, the end of the instrument is unavoidably moved from its position and the powder is thrown somewhere else than where intended. This objection does not apply to the insufflator which I show you. In using it, the glass tube, having been charged with the powder and introduced into the end of the flexible rubber tube, is held between the thumb and first finger of the right hand, and the rubber bulb is held in the palm of the same hand by the remaining fingers, so that it may be readily compressed without affecting the position of the glass tube. In charging the insufflator the end which is to be inserted into the rubber tube should be passed into the powder, and moved round and round until sufficiently filled ; it is then connected with the rubber tube. To make the application, if you cannot use the laryngoscope, hold the insufflator with your right hand and with the other hold the patient's tongue far out of the mouth, then direct the patient to take a deep inspiration, and during the act pass the tube as far back, and low down into the throat as you can, compress the bulb, and the powder will nearly always be blown directly into the larynx. The powder which will be most beneficial in cases of tuberculous ulceration consists of one grain of morphia to about forty grains of bismuth and ten grains of pulverized acacia ; or, if there is much secretion, you may substitute a portion of the bismuth by tannin or iodoform, or both. When the pain in deglutition is severe, you can give your patient the greatest relief by the application to the ulcer, by means of the laryngeal brush, of a solution of tannin and carbolic acid in glycerine ; I use four grains of morphia, thirty of tannin, and thirty of carbolic acid to the ounce of glycerine. The relief which I have been able to procure such cases by this application has given me more satisfaction than anything else during my professional life. In illustration, I will cite only a couple of cases : A gentleman called on me some time ago, in a most piteous condition, who, on account of the pain, had found it impossible to swallow for a week. Upon laryngoscopic examination, I found a large and deep ulcer on the lingual surface of the epiglottis. I brushed this solution well over the surface of the ulcer, and he returned to me the next day, having eaten his supper and breakfast without the slightest difficulty. I made another application, and then saw nothing more of the patient for about two months. On his return, he told me that he had not suffered in the least since the last application, until the last few days. I made another application, and the patient has not yet returned.

In another case, there was a large ulcer on the ventricular band of the left side, and extending to the inter-arytenoid fold ; the pain being so great on attempts to swallow, that the patient was unable to take either fluids or solids. I made an application which relieved the pain entirely, and made the patient comfortable for nearly two days. Subsequently the applications were made nearly every second day, for several weeks, each time giving the patient almost complete immunity from pain, for from forty to forty-eight hours, with the result of allowing the ingestion of food and drink, and prolonging life for a couple of months, until the patient succumbed to the constitutional disease.

